# Emergency Medical Services (EMS) Utilization in Zimbabwe: Retrospective Review of Harare Ambulance System Reports

**DOI:** 10.5334/aogh.3649

**Published:** 2022-08-12

**Authors:** Monalisa Muchatuta, Soman Mudariki, Loretta Matheson, Brian Rice, Midion Chidzonga, Rebecca Walker, Matthew Strehlow, Jennifer Newberry

**Affiliations:** 1 Stanford University School of Medicine, Department of Emergency Medicine, Palo Alto CA, USA; 2 SUNY Downstate Medical Center, Department of Emergency Medicine, Brooklyn NY, USA; 3 City of Harare Ambulance, Emergency Medical Services, Harare, ZW; 4 University of Zimbabwe, College of Health Sciences, Depart of Dentistry and Oral-Maxillofacial Surgery, Harare, ZW

**Keywords:** Zimbabwe, Africa, EMS, Ambulance, Global Health, Trauma, Obstetrics

## Abstract

**Background:**

Emergency medical services (EMS) are a critical but often overlooked component of essential public health care delivery in low- and middle-income countries (LMICs). Few countries in Africa have established EMS and there is scant literature to provide guidance for EMS growth.

**Objective:**

This study aimed to characterize EMS utilization in Harare, Zimbabwe in order to guide system strengthening efforts.

**Methods:**

We performed a retrospective chart review of patient care reports (PCR) generated by the City of Harare ambulance system for patients transported and/or treated in the prehospital setting over a 14-month period (February 2018 – March 2019).

**Findings:**

A total of 875 PCRs were reviewed representing approximately 8% of the calls to EMS. The majority of patients were age 15 to 49 (76%) and 61% were female patients. In general, trauma and pregnancy were the most common chief complaints, comprising 56% of all transports. More than half (51%) of transports were for inter-facility transfers (IFTs) and 52% of these IFTs were maternity-related. Transports for trauma were mostly for male patients (63%), and 75% of the trauma patients were age 15–49. EMTs assessed and documented pulse and blood pressure for 72% of patients.

**Conclusion:**

In this study, EMS cared primarily for obstetric and trauma emergencies, which mirrors the leading causes of premature death in LMICs. The predominance of requests for maternity-related IFTs emphasizes the role for EMS as an integral player in peripartum maternal health care. Targeted public health efforts and chief complaint-specific training for EMTs in these priority areas could improve quality of care and patient outcomes. Moreover, a focus on strengthening prehospital data collection and research is critical to advancing EMS development in Zimbabwe and the region through quality improvement and epidemiologic surveillance.

## Introduction

Many drivers of mortality in LMICs are time-sensitive conditions, such as sepsis, trauma, and postpartum hemorrhage, that are responsive to emergency care [1,2,3,4]. Consequently, in 2019 a World Health Assembly resolution recognized emergency care as an essential component of healthcare systems and recommended that member states comprehensively assess their prehospital and hospital-based emergency care functionality [5].

Emergency medical services (EMS) are a critical but traditionally overlooked component of emergency care systems and they are necessary for the expedient transport and care of patients from the field or to higher levels of care from other facilities [1,6]. Yet, only one third of African countries have EMS [7,8]. Currently in Africa, EMS ranges from basic transport by non-medical providers to advanced services with trained paramedics, including air transport [9]. Most EMS in Africa is a pay-for-service model, with some countries having varying combinations of private, government, and charity-run ambulance systems [10,11].

Zimbabwean EMS was first established in 1984 with private and public ambulance systems staffed by trained professionals ranging from basic emergency medical technicians (EMTs) to paramedics [11]. Until the mid-1990s, EMS in Zimbabwe boasted well-trained providers and state of the art equipment. However, Zimbabwe has experienced economic instability over the past 20 years, leading to poverty and declining health outcomes [12,13]. As the economy declined so did the state of EMS, which was compounded by a growing population unable to pay for EMS. This increased the demand for government and/or charity-run EMS over pay-for-service models. The economic strife has also made it extremely difficult for public EMS to maintain or acquire more modern equipment, resulting in the current stagnant state of EMS and healthcare more broadly [12]. As the country works to rebuild healthcare infrastructure, it is critical to understand the current state and the future potential of Zimbabwean EMS to improve health outcomes.

To date, there has been scant literature on Zimbabwean EMS and the data has only been by self-report [11,14]. There have been no studies on the epidemiology of emergency care conditions or the specifics of EMS utilization in Zimbabwe. In this study, we begin to address this critical gap in the literature. We conducted a retrospective review of patient care reports (PCRs) to characterize the utilization of EMS and associated transport patterns in Zimbabwean EMS system. This study is important for strengthening the developing emergency care system in Zimbabwe and for providing useful insights for other LMICs.

## Methods

### Study setting and design

The City of Harare Ambulance (COHA) system is the oldest and longest running EMS system in Zimbabwe, having been initiated in 1984. This ambulance service is locally run under city authority and budget. It is the only government-funded EMS system in Harare and is available free of charge to all 2.1 million residents [15]. The EMS landscape in Harare also has numerous private pay-for-service ambulance services whose patients are those that have access to insurance or have the financial ability to pay for the service. However, over 75% of all Harare residents lack health insurance, with the prevalence of uninsured highest among people in the bottom 3 wealth quintiles (>98%) [16].

COHA operates advanced life support-capable vehicles staffed with trained providers and hosts the only public training center in Harare for EMS providers. COHA trains ambulance technicians, EMTs, and paramedics, who can either stay with COHA or go on to work in the private sector. Ambulance technicians have 4 weeks of basic life support training. EMT training is 12 weeks, including advanced life support training and five weeks of supervised practice. Paramedics are the most highly trained ambulance provider, with 30 weeks of advanced life support training.

We conducted a retrospective review of patients who were transported and/or treated by COHA during the 14-month period from February 2018 to March 2019. For every EMS transport, ambulance providers record basic patient data and operation metrics on a standardized instrument, a patient care report (PCR). All PCRs are written in English on paper forms. Due to archiving methods, many PCRs have been damaged or lost over years. We reviewed all available PCRs from our study period.

Ethical approval for this study was provided by the Stanford University Institutional Review Board (Palo Alto, California, USA; #50206), the City Council of Harare (Harare, Zimbabwe), and the Medical Research Council of Zimbabwe (Harare, Zimbabwe; #MRCZ/E/257).

### Data collection

We created a standard electronic data collection instrument that mirrored COHA’s paper PCR forms. The paper PCR forms included several specific fields, including patient demographics, pickup location, incident type, patient vital signs, and patient condition on handover at the receiving facility. We created 5 additional fields to capture specific data the EMT may have noted in an area of the PCR with free text narrative: destination hospital; intervention by EMT; events during transport; impression by EMT; and a binary field indicating whether or not the EMT had performed a physical examination. For each of these, the lists were based on an initial review of the narratives and refined as new items were identified in the data. Two research assistants, with experience as COHA paramedics, were trained to extract study variables from the handwritten PCR forms. All data were securely collected and managed via REDCap (Stanford University) [17].

### Data analysis

We conducted a descriptive analysis of patient demographics, chief complaints, and transport patterns, using descriptive statistics, providing frequencies and percentages as appropriate. Chief complaints were recorded as free text in the PCRs. Consequently, we chose to group chief complaints into 10 broad chief complaint categories using the categorization from Mould-Millman et al. [14] as a framework, which was chosen because of its regional relevance. Chief complaint categorization was completed by the lead author and then reviewed by the senior author to reach consensus. COHA also categorized transports by incident type. Incident type is an internally designated classification of the different call types that COHA receives. The dispatcher determines incident type based on COHA’s predetermined grouping: maternity, medical, road traffic accident (RTA) and other accident or assault. This is distinct from chief complaint in this study as chief complaints were determined by the patient and EMT. All data analysis was conducted in SAS Enterprise Guide for Windows, V.4.3 (SAS Institute, Cary, North Carolina, USA).

## Results

A total of 875 PCRs from the COHA archives were reviewed. Based on dispatch logs from the study period, we estimate the PCRs reviewed here represent approximately 8% of calls resulting in dispatches that were not otherwise cancelled (e.g. patient not found, patient cancellation). The majority of patients were age 15–49 (76%, n = 668) (Table 1). Notably, slightly more than half of the EMS use was for inter-facility transfers (IFTs) rather than transports from the community. IFTs initiated from healthcare facilities, whether clinic or hospital. Most IFTs were labeled as ‘maternity’ incidents (52%, n = 228) and female patients constituted 78% (n = 344) of all IFTs. It should be noted that all pregnant patients who have a traumatic injury are still labelled with an incident type of “Maternity” because all of these women are taken to hospitals with obstetric care capacity. Pick-ups from the community were mostly trauma-related (69%), these transports from the field were predominantly for male patients (61%, n = 200).

**Table 1:** Patient Information by Inter-facility Transfer or Pickup in the Community.


TOTAL	TOTAL		INTER-FACILITY TRANSFER (IFT)		PICKUP IN COMMUNITY	
		
	N		N	%	N	%
		
	875		442	51%	328	37%

**Incident Type**						

Road-Traffic Accident	**262**	30%	19	4%	227	69%

Maternity	**254**	29%	228	52%	6	2%

Medical	**227**	26%	149	34%	51	16%

Other Accident or Assault	**62**	7%	24	5%	34	10%

**Gender**						

Female	**532**	61%	344	78%	113	34%

Male	**323**	37%	95	21%	200	61%

**Age**						

0–14 years	**47**	5%	27	6%	14	4%

15–49 years	**668**	76%	359	81%	235	72%

50–64 years	**51**	6%	18	4%	26	8%

65+ years	**42**	5%	15	3%	20	6%

**Vital signs were abnormal – % of recorded**						

Tachycardic (of N = 645)	**188**	29%	110	36%	56	21%

Hypotensive* (of N = 737)	**25**	3%	11	3%	10	3%

Hypertensive (of N = 737)	**39**	5%	18	5%	18	6%

Hypoxic (of N = 508)	**42**	8%	12	6%	21	8%

Altered Level of Consciousness† (of N = 739)	**95**	13%	35	10%	49	17%


* Systolic < 100 or Diastolic < 50. † GCS < 15. Missing: Incident type 8%, Pickup type 12%, Gender 2%, Age 8%.

Trauma and pregnancy were the most common chief complaints overall, comprising 56% of all calls (n = 484) (Table 2). Trauma was the predominant complaint in men (63%, n = 153) and mainly in the age group 15–49 (75%, n = 182).

**Table 2:** Chief Complaint by Gender and Age Range.


TOTAL	TOTAL		GENDER				AGE RANGE							
	
			FEMALE		MALE		0–14 YEARS		15–49 YEARS		50–64 YEARS		65+ YEARS	
					
	N	%	N	%	N	%	N	%	N	%	N	%	N	%
					
	875		532	61%	323	37%	47	5%	668	76%	51	6%	42	5%

**Chief Complaint**														

Trauma	**242**	28%	81	33%	153	63%	11	5%	182	75%	23	10%	7	3%

Pregnancy	**242**	28%	242	100%	0	–	0	–	237	98%	0	–	0	–

Gastrointestinal	**82**	9%	57	70%	23	28%	7	9%	62	76%	5	6%	5	6%

Headache	**59**	7%	27	46%	30	51%	4	7%	45	76%	2	3%	1	2%

Cardiac	**54**	6%	26	48%	26	48%	4	7%	31	57%	7	13%	6	11%

Altered Mental Status	**37**	4%	10	27%	24	65%	4	11%	19	51%	1	3%	5	14%

Malaise	**24**	3%	14	58%	10	42%	1	4%	12	50%	4	17%	6	25%

Respiratory	**22**	3%	12	55%	10	45%	6	27%	10	45%	2	9%	3	14%

Neurologic	**21**	2%	15	71%	6	29%	0	–	18	86%	0	–	1	5%

Other*	**19**	2%	13	68%	5	26%	1	5%	13	68%	1	5%	1	5%


* Other: Fever, Jaundice, Urologic, Mental health Missing: Chief Complaint 8%, Gender 2%, Age 8%.

EMT assessment forms were reviewed for documented vital signs. We found that EMTs recorded both pulse and blood pressure for 72% of patients (n = 628) but less than half of the forms had all vital signs documented (Table 3).

**Table 3:** EMT Assessments.


TOTAL	N	%

	875	

**Vital signs were recorded**		

Pulse	**645**	**74%**

Blood pressure	**737**	**84%**

Oxygen saturation	**508**	**58%**

Mental status	**739**	**84%**

Both Pulse and Blood pressure were recorded	**628**	**72%**

All four of the above vitals were recorded	**419**	**48%**


## Discussion

This study represents the first retrospective chart review of EMS in Zimbabwe and adds to the body of literature on the requirements of EMS in Africa. For Zimbabwe, this study can provide an initial framework for clinical, operational, and public health efforts surrounding EMS. We found that over half of EMS transports were for IFTs. This is consistent with research on similar ambulance systems in Sub-Saharan Africa or 45% IFTs in Ghana’s National ambulance system EMS assessment [14]. Strikingly, maternity-related transports accounted for slightly over half of all IFTs, which is internally consistent with the finding that the IFT patients were primarily women of child bearing age presenting with a pregnancy-related chief complaint. Prior research across LMICs has also shown that obstetrics cases often account for a large proportion of emergency care transports in developing EMS systems [18].

The high percent of maternity-related IFTs may be related to low numbers of comprehensive emergency obstetric care (CEmOC) centers, where blood transfusions and surgeons are available to perform caesarean sections [19]. While LMICs have long been encouraged to promote births in CEmOC facilities, these centers may be few and their costs prohibitive [20]. A large proportion of women in LMICs rather delivers in midwife-led facilities without advanced obstetric providers or comprehensive emergency resources available [20,21]. When obstetric emergencies do arise, women from remote areas may face a lack of transport to higher levels of care from remote areas [21]. This then results in extended travel times when expediency is most needed to prevent complications to newborn (e.g., asphyxia, perinatal death) and mother (e.g., vaginal fistula, peripartum bleeding) [22]. In countries like Zimbabwe where EMS is growing, this burden can be mitigated by leveraging EMS to transfer pregnant patients between facilities as seen in our study. COHA ambulances being the only government-owned EMS in the city are the main health service transporter of patients to public hospitals. COHA ambulances will pick up patients from any location including government/private clinics or hospitals and deliver all patients to one of two public hospitals in Harare depending on proximity: Parirenyatwa or Harare Hospital. Other LMICs have shown that EMS can transport rural obstetric patients to needed emergency care in under 2 hours, an internationally recommended metric [20]. Our findings demonstrate that COHA is already an active component of the public health infrastructure for obstetric emergencies, further integration with CEmOC centers and expansion of this lifeline to more rural settings are needed.

The second equally common chief complaint in our study was trauma. Our study demonstrated that the majority of trauma-related EMS calls to COHA were for men (63%) and for the age range 15–49 years (75%). These findings are consistent with previous research on the epidemiology of trauma in LMIC [23]. LMICs account for approximately 90% of all global cases of trauma-associated deaths [24]. Globally, road traffic injuries contribute to the high rate of trauma-related morbidity and mortality in LMICs and have become a sustained development goal target [25,26]. Our study showed a predominance of trauma-related chief complaints at 28% of total chief complaints (Table 2). This finding suggests a major need for competent trauma specific emergency services. However, across numerous African countries, patients with emergency traumatic conditions are often limited in their ability to directly access higher level hospitals [9,10,14,27]. Similar to maternity patients, a lack of rapid transport is a critical barrier to timely trauma care, and correspondingly, to improving outcomes [24,28]. The availability of prehospital care services has been estimated to lead to a 25% mortality reduction in traumatic injured patients [29]. Further growth and strengthening of EMS in Zimbabwe could alleviate this barrier and help combat the burden experienced from injuries.

The utilization of EMS transport by COHA is similar to that reported by other EMS systems within the African region [14]. Similar to other systems, we saw that IFTs were more common than field transports and that pregnancy-related and trauma chief complaints were the most common amongst transports. We postulate that opportunities for system strengthening align with Mould-Millman et al. study in Ghana, which advocated for reliable revenue, increased public access, community integration, research, and improved quality assurance processes as a way to continue to develop EMS [27].

To strengthen EMS and respond to the growing demand for high-quality prehospital care, PCRs can provide a method for EMT assessment and quality improvement. Assessing the PCRs, we found that EMTs in COHA recorded a full set of vitals (pulse, blood pressure, oxygen saturation, and mental status) only about half the time (48%). This finding may represent that either the EMT has not assessed all of the patient’s vital signs or that they simply failed to record their findings. An emphasis on improving the EMTs use of PCRs is required, and may be strengthened by adding options to the PCR such as “unable to assess blood pressure”. Additionally, while not present in this study, metrics on call times and transport times could be added to PCRs and would provide important quality improvement targets. COHA has set a goal for increased quality in data recording and strengthening archive methods, both of which will increase the usefulness of PCRs as a backbone for quality improvement for EMS in Harare.

### Limitations

The largest limitation of this study was that few PCRs were available compared to the actual number of transports conducted. As noted above, even though we reviewed all available PCRs in our study period, we estimate this accounts for less than 8% of the total transports conducted during this period. Given the limited information recorded per call in the dispatch logbooks, it is unclear whether the PCRs that are archived are a random sample or may be skewed. Nevertheless, we believe this preliminary descriptive analysis of the available data remains useful and gives insights into the range of chief complaints seen, the types of transports conducted, and the EMTs use of the PCRs. COHA is currently seeking to change its archiving methods.

PCR forms also introduced some limitations during formation of the data collection instrument. About 25% of the PCR were written in free text, making translation into a standardized data collection instrument difficult. However, most of the information in the free text area was recorded elsewhere on the form, minimizing the impact of this weakness. Lastly, absence of previous EMS data in the country did not allow chief complaints to be categorized based on locally contextualized evidence-based groupings, and rather we relied on categorizes outlined by regional patterns [14,30].

## Conclusion

The majority of transports by COHA EMS in Zimbabwe are inter-facility transfers, half of which are obstetrics chief complaints. Overall, this EMS system cares primarily for obstetric chief complaints and traumatic injuries, which mirrors the leading causes of premature death in LMICs. COHA is the largest government-funded EMS system in Harare catering to the majority of underprivileged people. Further efforts are needed to integrate and strengthen COHA and other ambulance systems in the country with the public health infrastructure for both trauma and obstetric emergencies. Moreover, a focus on prehospital data collection for quality improvement and epidemiologic surveillance can serve to advance EMS development in Zimbabwe and the region through research and supporting quality improvement processes.
